# Integrated Assessment of Behavioral and Environmental Risk Factors for Lyme Disease Infection on Block Island, Rhode Island

**DOI:** 10.1371/journal.pone.0084758

**Published:** 2014-01-08

**Authors:** Casey Finch, Mohammed Salim Al-Damluji, Peter J. Krause, Linda Niccolai, Tanner Steeves, Corrine Folsom O’Keefe, Maria A. Diuk-Wasser

**Affiliations:** 1 Department of Epidemiology of Microbial Diseases at Yale School of Public Health, New Haven, Connecticut, United States of America; 2 Department of Internal Medicine at Yale School of Medicine, New Haven, Connecticut, United States of America; 3 Audubon Connecticut, Southbury, Connecticut, United States of America; Fondazione Bruno Kessler, Italy

## Abstract

Peridomestic exposure to *Borrelia burgdorferi*-infected *Ixodes scapularis* nymphs is considered the dominant means of infection with black-legged tick-borne pathogens in the eastern United States. Population level studies have detected a positive association between the density of infected nymphs and Lyme disease incidence. At a finer spatial scale within endemic communities, studies have focused on individual level risk behaviors, without accounting for differences in peridomestic nymphal density. This study simultaneously assessed the influence of peridomestic tick exposure risk and human behavior risk factors for Lyme disease infection on Block Island, Rhode Island. Tick exposure risk on Block Island properties was estimated using remotely sensed landscape metrics that strongly correlated with tick density at the individual property level. Behavioral risk factors and Lyme disease serology were assessed using a longitudinal serosurvey study. Significant factors associated with Lyme disease positive serology included one or more self-reported previous Lyme disease episodes, wearing protective clothing during outdoor activities, the average number of hours spent daily in tick habitat, the subject’s age and the density of shrub edges on the subject’s property. The best fit multivariate model included previous Lyme diagnoses and age. The strength of this association with previous Lyme disease suggests that the same sector of the population tends to be repeatedly infected. The second best multivariate model included a combination of environmental and behavioral factors, namely hours spent in vegetation, subject’s age, shrub edge density (increase risk) and wearing protective clothing (decrease risk). Our findings highlight the importance of concurrent evaluation of both environmental and behavioral factors to design interventions to reduce the risk of tick-borne infections.

## Introduction

Lyme disease, caused by the spirochete *Borrelia burgdorferi*, is the most commonly reported vector-borne disease in the US, with greater than 20,000 cases reported annually [1]. The black-legged tick (*Ixodes scapularis*) serves as the principal vector for transmission to humans and is responsible for maintenance of the spirochete in natural reservoirs. Since the Lyme disease vaccine was removed from the market in 2002 [2], strategies to reduce the number of human cases of Lyme disease have focused on ways to control ticks and pathogens in zoonotic hosts through host-targeted acaricides [3], [4] and host vaccination [5], [6], or to help decrease contact between humans and infected ticks. The latter approach has consisted of either reducing the density of *I. scapularis* nymphs infected with *B. burgdorferi* (acarological risk) through area-wide acaricides [7]–[9], reducing human-tick contact through environmental management [10], or the use of personal protective measures [11]–[13]. These methods that modify behavior or the environment vary in their effectiveness in reducing human disease and there is no consensus on which ones should be emphasized [14]–[16].

Attempts to identify the best targets for intervention have been hindered by the disparity in approaches, in spatiotemporal scales and in the level of analysis of studies focusing on acarological, landscape or behavioral risk factors. A link between Lyme disease incidence and acarological risk was found at the aggregate town or county level [17]–[23]. At a neighborhood scale, landscape features have been linked to increased acarological risk [24]–[27], but the direct link between acarological risk and Lyme disease was found to be weak [27]. The latter study did not assess human behaviors, which can modify the association between acarological risk and human infection. On the other hand, studies of the effectiveness of human protective behaviors were conducted by telephone interviews and did not directly measure landscape patterns or acarological risk [28], [29]. These studies may underestimate the effectiveness of these protective behaviors if residents of high acarological risk properties are more likely to perform them.

Logistical challenges are likely responsible for the lack of integrated acarological, landscape, and behavioral studies. Measuring acarological risk in a large number of properties is difficult because of the short window of *I. scapularis* nymphal activity between late May and early July [30]. Another limitation of previous studies is the reliance on human clinical cases as the health outcome. Reports of clinical cases only identify symptomatic infections that are diagnosed and reported, which are only a portion of the actual number of infections [31]–[33].

In the present study, we simultaneously assessed the association between Lyme disease and both individual and environmental risks on Block Island, RI, between 2005 and 2011. High resolution imagery was used to measure the amount and configuration of lawn-shrub edges in all residential properties, where most human-tick contact is expected to occur. This measure of environmental risk was validated by collecting ticks along those edges on a subset of properties. A sensitive measure of human infection was obtained through serosurveys conducted twice a year, where personal protective behaviors and *B. burgdorferi* seroprevalence was assessed. The integration of behavioral and environmental risk assessments allows more accurate identification of intervention targets by controlling for modifying factors and minimizing confounding.

## Methods

### Study Site

Block Island is a 25.2 km^2^ landmass located in Washington County, Rhode Island, 23 km south of mainland Rhode Island [34]. The population of permanent residents is around 1,000, which increases during the summer months to approximately 12,000 with the influx of summer residents [31]. Deciduous forest, the most suitable habitat type for *Ixodes scapularis* in the mainland, is limited on the island to a 4 ha site [35], so most tick habitat on the Island is restricted to shrublands and shrub edges with sufficient leaf litter accumulation for tick survival.

### Study Population

A longitudinal study was established in 1991 on Block Island, RI, by inviting all island residents to take part in serological surveys conducted every year in the spring and fall. The study population was restricted to residents who spent more than one month on the island during the May through September Lyme disease transmission period. The serosurvey was announced in the local newspaper, on television, and via flyers at local businesses and the Block Island Medical Center [31], [36]. All subjects were asked to provide blood samples for *B. burgdorferi* and *B. microti* serological analyses and to complete a questionnaire. Written informed consent was obtained from all study participants in accordance with the human investigation committees at the University of Connecticut School of Medicine and the Yale School of Public Health. They each provide ethical review and oversight of human research endeavors and approved this study.

We restricted our analysis to subjects who participated in serosurveys from 2005 to 2011 because environmental exposure was assessed based on a 2010 satellite image. Landscape metrics were assumed to be minimally changed during that period. To minimize the influence of a recent Lyme disease diagnosis on an individual’s behavior, we excluded from the study individuals reporting a Lyme disease diagnoses within two years of their initial serosurvey visit and data from all subsequent visits after an individual developed positive *B. burgdorferi* serology.

### Lyme Disease Exposure Definition

We defined a person with Lyme disease exposure as an individual who tested positive for *B. burgdorferi* antibody using a standard two tier ELISA and western blot antibody approach [36]. A positive ELISA result consisted of an IgM or IgG response at ≥1∶320 dilution. Positive or equivocal ELISA results were confirmed by western blotting. Specimens were considered positive if 5 or more bands of the ten most prevalent *B. burgdorferi*-specific bands were present in the immunoblot [36]–[38]. All antibody assays prior to the fall of 2008 were carried out at the University of Connecticut Health Center. Assays from the spring survey of 2009 until the fall of 2011 were performed by commercial laboratories in New England using standard Lyme serodiagnostic assays.

### Individual Risk Factor Assessment

All subjects were asked to complete a questionnaire assessing the history of previous tick borne illnesses, peridomestic factors potentially linked to tick exposure, their age, protective behaviors and outdoor activities (Table 1, Figure S1). The questionnaire was administered at the time of the blood draw and included questions about regularly performed behaviors related to tick exposure.

**Table 1:** Behavioral and demographic characteristics of survey responders in relation to their serological status.

Variable	Seropositive	Seronegative	Total
Hours spent in vegetation	2.20 hours (85)	2.01 hours (817)	2.03 hours (902)
Owning a dog	39.24% (79)	36.86% (738)	37.09% (817)
Owning a cat	31.65% (79)	26.59% (737)	27.08% (816)
Owning a horse	0% (79)	0.54% (737)	0.49% (816)
Owning a different pet	6.33% (801)	3.53% (736)	3.80% (815)
Tick bite within the past year	28.57% (84)	25.66% (799)	25.93% (883)
Tick bite within the past year on Block Island	29.51% (61)	27.53% (534)	27.73% (595)
Use of any protective measure*	66.67% (84)	73.59% (814)	72.94% (898)
Repellent	11.84% (76)	17.06% (768)	16.59% (844)
Protective clothing	27.63% (76)	46.09% (768)	44.43% (844)
Avoiding brush	32.89% (76)	38.64% (766)	38.12% (842)
Tick checking	47.37% (76)	53.84% (769)	53.25% (845)
Landscape-related tick control measures	24.71% (85)	24.97% (809)	24.94% (894)
Area-wide acaricide use	0 (67)	1.65% (665)	1.50% (732)
Previous Lyme diagnosis	85.90% (78)	33.28% (640)	39.00% (718)
Shrub percentage of land	36.58% (86)	31.94% (809)	32.39% (895)
Occupational exposure to tick habitat	7.69% (78)	11.43% (761)	11.08% (839)
Age at the test	66.13 years (86)	61.02 years (802)	61.52% (888)

Percent positive (or average) responses over the total responses for each question for behaviors and age reported by *B. burgdorferi* seropositive and seronegative participants in serological surveys between 2005 and 2011.

Use of any protective measure = use of either protective clothing, tick checking, repellent or avoiding brush.

### Environmental Risk Assessment

We developed remotely sensed landscape metrics that quantified the amount of edge between lawn and shrub vegetation at all subject residences. We hypothesized that these sites would be areas of high tick density and increase human contact with ticks. We first generated a high resolution land cover classification of Block Island, and then calculated the composition and configuration (landscape metrics) of shrub and lawn cover characteristics for each individual property. Finally, we assessed the association between the landscape metrics and the density of *I. scapularis* nymphs in a representative subset of properties, as described below.

#### Land cover classification

**Figure 1:**
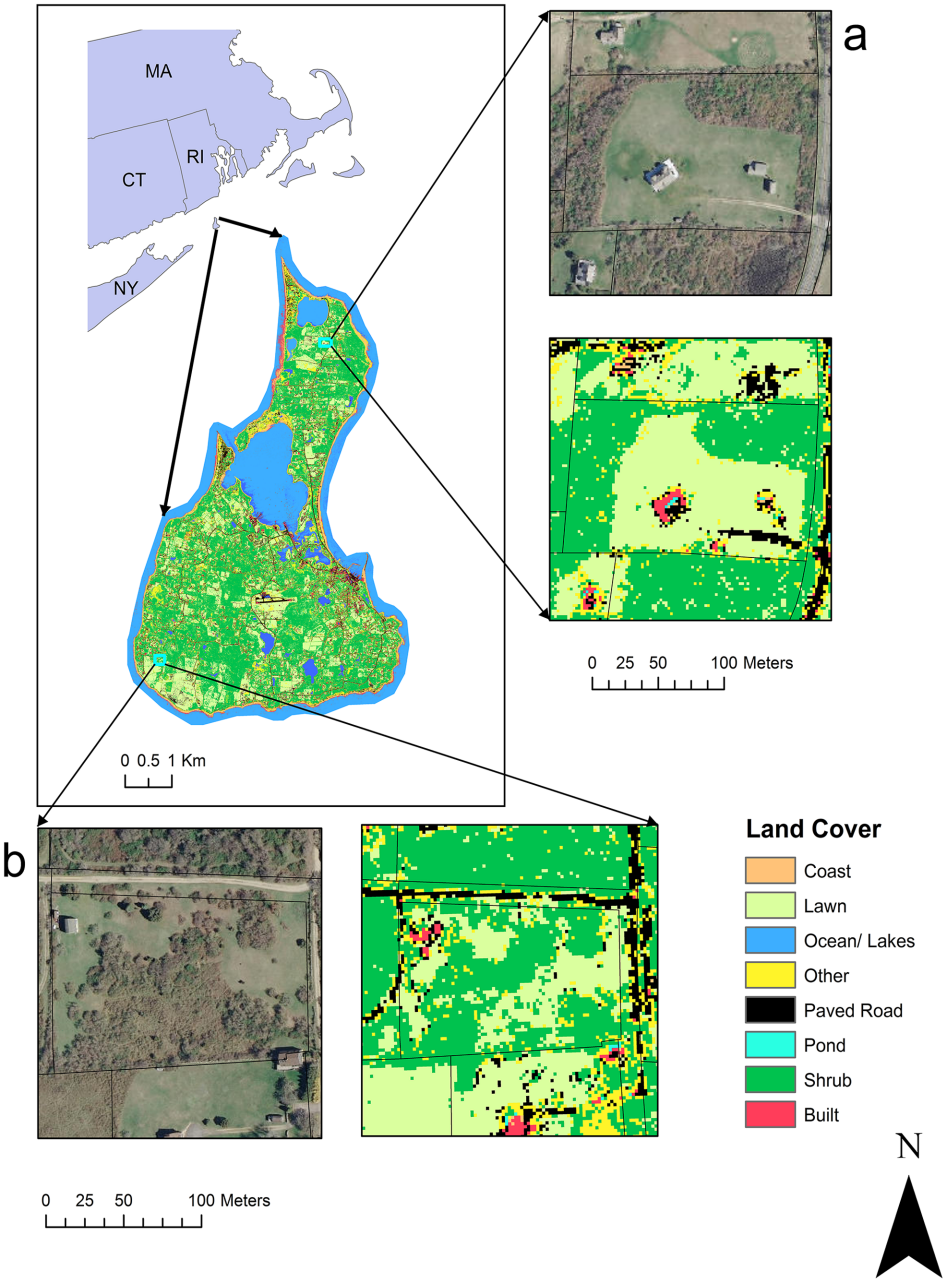
Land cover classification of Block Island, Rhode Island. Examples of properties with a) low shrub edge density and b) high shrub edge density. Map shows a Worldview2 image acquired on Sept. 4, 2010 from Digital Globe, Inc.

We generated a land cover classification of Block Island using WorldView2 satellite sensor data acquired on September 10, 2010. WorldView2 has a spatial resolution of 1.82 m that allows detection of fine scale peridomestic landscape patterns. It also has high spectral resolution (8 bands), resulting in greater ability to discriminate among land cover types than the four bands typically available for other high spatial resolution sensors. We converted the data to top of the atmosphere radiance [39]. We performed a maximum likelihood land cover classification using ENVI (Environment for Visualizing Images) software (ITT 2011) [40]. To improve the accuracy of the classification, the image was stratified into vegetated and non-vegetated areas based on a threshold of the normalized difference vegetation index = 0 [40] and the classification was performed independently for each stratum. Training and testing pixels were obtained by collecting ground information for all vegetation classes and by visual inspection of a 2010 orthophoto for the water and urban-associated classes, which easily could be distinguished. A randomly selected subset of 80% of the pixels was used to train the classification and 500 pixels were randomly selected from the remaining 20% for each of the classes for testing. After the classification, the two strata were combined in one raster layer and a 3×3 pixel median filter was applied to remove the salt and pepper effect (Figure 1).

#### Landscape metric calculation

Spatial analysis was performed utilizing geo-spatial modeling environment (GME) software version 0.5.8 beta [41] and Fragstats software version 3.3 [42]. We used a municipal parcel layer (Town of New Shoreham) to calculate the following landscape metrics for shrubs and lawns in each property parcel: area of the landscape class, largest patch index, total edge, edge density and landscape shape index (calculations described in Table S1). Landscape metrics were normalized using the Z-scale ([X-mean]/standard deviation) prior to use in statistical analyses.

#### Association between landscape metrics and the density of *I. scapularis* nymphs

During 2012, *I. scapularis* nymphs were collected from 105 properties of serosurvey participants from May 15^th^ to August 23^rd^. The property surveys consisted of dragging 1 m^2^ corduroy cloths along the edge of the lawn and shrub vegetation as outlined in previous studies [43]–[45]. Between 2 and 5 transects of approximately 100 meters in length were completed at each property, proportionally to the size of the property. Most properties were repeatedly sampled during the season, resulting in a total of 258 samples. Attached *I. scapularis* nymphs were counted, placed in 70% ethanol, and species confirmed using taxonomic keys [46]. We based our measure of risk only on the density of host-seeking *I. scapularis* nymphs (hereafter density of nymphs) without calculating the proportion infected with *Borrelia burgdorferi* because the small number of nymphs collected on most properties prevented an accurate estimate of infection prevalence.

### Statistical Analyses

#### Identification of environmental risk factors

Negative binomial regression was used to assess the association between landscape metrics and the density of nymphs. Only those landscape metrics found to be significantly associated with the density of nymphs on a property were considered biologically relevant and thus included as potential risk factors in further analyses.

#### Individual and environmental risk factors for Lyme disease

We used general estimating equation models (XTGEE) in STATA/SE, version 12.0 (STATA Corporation, College Station, TX) to assess the association between personal protective behaviors, age, landscape metrics and individual serological status. These models fit generalized linear models that yield logistic regression models via a Bernoulli distribution of the dependent variable and a logit link function. The models accounted for potential autocorrelation among observations in a time series - in this case serological tests at different time periods on the same subject.

We performed univariate analyses for all variables and then examined multivariate models including all possible combinations of variables found to be significant in univariate analyses. We assessed two groups of models: one including self-reported Lyme disease diagnosis and one excluding this variable. Including self-reported Lyme disease is informative in terms of the consistency between previous and current risk; excluding this variable allowed for identification of current risk factors for Lyme disease infection. The maximum model size was reached when larger models resulted in all non-significant variables. We included age in all models to control for confounding. No more than one landscape metric was included in a model because these variables were highly collinear. Pairwise correlation among all variables was assessed and only variables with Pearson correlation coefficient lower than 0.2 were included in the same model. Models were compared by the QIC criterion, which an extension of the Akaike information criterion (AIC) [47]–[48] used for generalized estimating equation models [49]. The QIC is a measure of the relative quality of a statistical model for a given set of data. Similar to AIC, QIC not only rewards goodness of fit, but also includes a penalty that is an increasing function of the number of estimated parameters, resulting in the most parsimonious model [50]. We additionally assessed whether inclusion of variable interactions improved model fit and assessed the goodness-of-fit of the final model using the Hosmer-Lemeshow test [51], [52]. Finally, to determine whether there were spatial relationships among the properties not captured by the measured variables, we evaluated whether the residuals of the model were significantly autocorrelated using the *Moran’s I* test included in the ArcGIS Spatial Statistics toolbox [53], [54].

## Results

### Study Subjects

Of 611 subjects participating in at least one serosurvey between 2005 and 2011, blood samples and completed questionnaires were available from 520 (1132 records). In order to obtain data from subjects whose behavior was not potentially altered by knowledge of recent Lyme disease, 34 participants (86 records) were excluded because of a self-reported Lyme diagnosis in the two years prior to their enrollment in the study, resulting in a dataset of 486 participants (1046 records). Additionally, 136 subsequent records were excluded after subject’s developed positive *B. burgdorferi* serology, resulting in a final set of 486 participants (910 records).

The *B. burgdorferi* seropositivity rate from all blood samples was 9.5% (86/910). The average age of the participants at the testing date was 61.5 (SD 16.5) years. The use of any form of tick protection was reported by 72.9% of the study participants. Routine tick checks were the most commonly used protective measures (53.3%), while use of repellant was practiced the least (16.6%). A summary of behaviors reported by seronegative and seropositive participants is provided in Table 1.

### Identification of Environmental Risk Factors

Land cover classification accuracy was 83.6% and the Kappa coefficient was 0.82. The class-specific accuracy metrics are reported in Table S2. Two lawn-associated metrics evaluated were significantly and negatively associated with the density of nymphs, while all shrub-associated metrics were positively associated with the density of nymphs (Table 2). A total of 373 nymphs were collected in 166 transects; with an average collection of 4.9 (SD 23.5) nymphs per 100 m transect.

**Table 2:** Landscape predictors of the density of host-seeking *I. scapularis* nymphs.

Landscape Metric	Coefficient	P-value
Lawn Class Area	−0.383	0.572
**Lawn Largest Patch Index**	−**0.357**	**0.005**
Lawn Total Edge	0.113	0.715
**Lawn Edge Density**	−**0.347**	**0.022**
Lawn Landscape Shape Index	0.285	0.143
**Shrub Class Area**	**1.348**	**0.021**
**Shrub Largest Patch Index**	**0.422**	**0.018**
**Shrub Total Edge**	**0.857**	**0.012**
**Shrub Edge Density**	**0.486**	**0.002**
**Shrub Landscape Shape Index**	**0.485**	**0.002**

Negative binomial regression univariate models of the association between lawn and shrub landscape metrics and the density of host-seeking *Ixodes scapularis* nymphs (statistically significant results at p<0.05 are indicated in bold).

### Individual and Environmental Risk Factors for Lyme Disease

**Figure 2:**
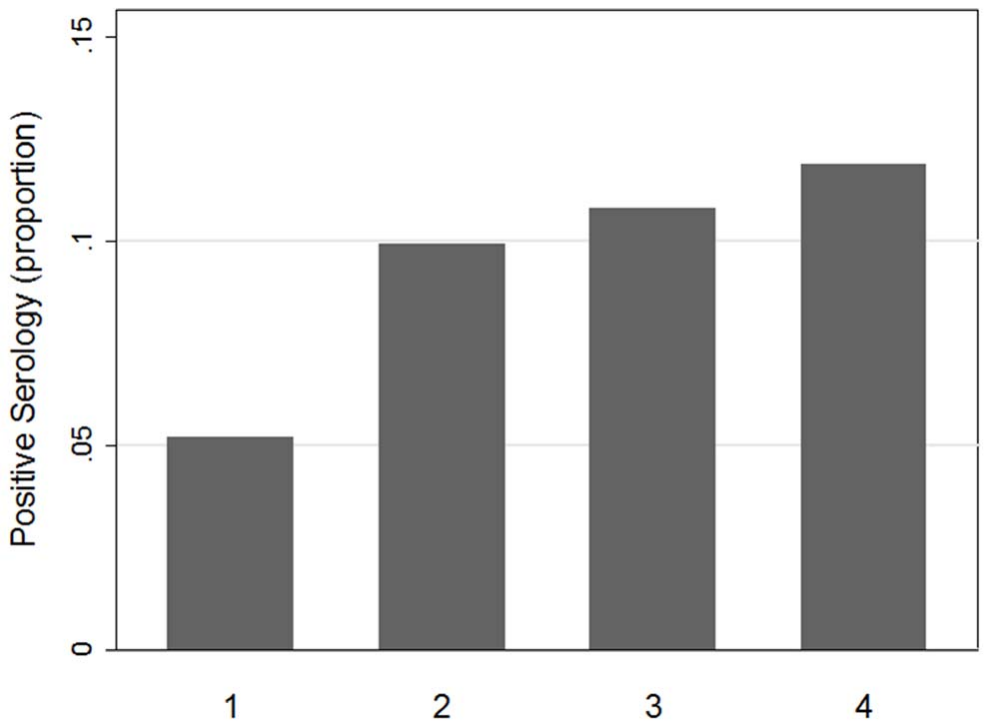
Proportion of seropositive subjects living in properties with increasing shrub edge density. Residences are classified into quartiles based on increasing shrub edge density.

Factors significantly associated with positive Lyme disease serology in univariate models included the average number of hours spent outdoors near vegetation, age at the time of testing, a self-reported previous Lyme disease diagnosis three or more years before testing and shrub edge density (Figure 2). Wearing protective clothing was significantly associated with negative Lyme serology (Table 3).

**Table 3:** Univariate models.

Variable	OR (95% CI)	QIC
**Age at the test**	**1.024 (1.002**–**1.046)**	**562.28**
**Previous Lyme diagnosis**	**7.437 (4.419**–**12.515)**	**426.69**
**BEHAVIOR**		
**Hours in vegetation**	**1.469 (1.081**–**1.996)**	**562.22**
Owning a dog	1.149 (0.719–1.835)	
Owning a cat	1.294 (0.788–2.125)	
Owning a different pet	1.960 (0.826–4.649)	
Frequency of deer seen on property	0.976 (0.727–1.310)	
Tick bite within the past year	1.111 (0.737–1.674)	
Tick bite within the past yearon Block Island	1.136 (0.659–1.960)	
Use of any protective measure	0.726 (0.454–1.161)	
Repellant	0.683 (0.337–1.386)	
**Protective clothing**	**0.456 (0.273**–**0.761)**	**505.14**
Avoiding brush	0.801 (0.490–1.309)	
Tick checking	0.786 (0.495–1.247)	
Landscape-related tickcontrol measures	1.001 (0.605–1.654)	
Occupational exposureto tick habitat	0.639 (0.263–1.556)	
**LANDSCAPE**		
Lawn largest patch index	0.899 (0.699–1.156)	
Lawn edge density	1.009 (0.809–1.258)	
Shrub class area	1.152 (0.941–1.410)	
Shrub largest patch index	1.204 (0.965–1.503)	
Shrub total edge	1.109 (0.938–1.310)	
**Shrub edge density**	**1.283 (1.015**–**1.621)**	**566.68**
Shrub landscape shape index	1.158 (0.944–1.419)	

Univariate logistic regression models of the association between human behaviors and landscape metrics and positive Lyme disease serology. Statistically significant results at p<0.05 are indicated in bold.

A multivariate model that included a previous Lyme disease diagnosis three or more years prior to recruitment and the subject’s age had the lowest QIC (423.88). When we excluded previous Lyme disease diagnosis from the analyses, the best fit multivariate model (QIC = 481.64) included hours spent outdoors near vegetation, shrub edge density and age (increased risk) and wearing protective clothing (decreased risk) (Table 4). Excluding either the landscape or behavioral factors from this model resulted in increases of QIC of more than two units, which indicate a significantly worse fit to the data [51]. There were no significant models including any combination of five or more variables. Inclusion of variable interactions did not improve model fit. The Hosmer-Lemeshow test indicated a good model fit (p = 0.17) and the *Moran’s I* test on the logistic regression residuals showed no significant autocorrelation (p = 0.38).

**Table 4:** Multivariate model.

	OddsRatio	SE	Z-score	P-value	95% CI	95% CI
					(Lower)	(Upper)
Hours in vegetation	1.735	0.308	3.10	0.002	1.224	2.460
Protective clothing	0.413	0.108	−3.38	0.001	0.247	0.689
Shrub edge density	1.315	0.182	1.98	0.048	1.002	1.725
Age at test	1.033	0.013	2.48	0.013	1.006	1.060
Constant	0.005	0.006	−4.78	0.000	0.001	0.047

Best fit (lowest QIC score) multivariate logistic regression model of the association between human behaviors and landscape metrics and positive Lyme disease serology.

## Discussion

Our study emphasizes the need for integrated studies of both environmental and behavioral risk factors to identify Lyme disease intervention targets. The best fit model for Lyme disease included two behaviors that consisted of hours spent in vegetation (increased risk) and wearing protective clothing (decreased risk), and an environmental factor that consisted of a landscape metric quantifying the density of edge between shrubs and other land use classes, particularly lawn, where human exposure to ticks is more likely to occur (increased risk). Higher density of shrub edge was positively associated with the density of nymphs, supporting this habitat as a source for human infection. Although human risk of infection with Lyme disease was best predicted by a self-reported previous Lyme disease diagnosis, we excluded this variable from further analyses to gain insights into specific behavioral and environmental risk factors. The strength of the previous Lyme disease association suggests that the same sector of the population tends to be repeatedly infected, either because of their behavior or their environmental exposure or both. These data also suggest that people at high risk of Lyme disease remain at high risk over time.

The first line of defense in the effort to prevent Lyme disease is personal protective behavior [14]. Consistent with previous studies [29], wearing protective clothing was a significantly protective behavior against Lyme disease. We did not identify a significant effect of tick checks, which was found to be effective in one previous study [28] but not in another [29]. Notably, despite the potential protective value of these methods and the high prevalence of Lyme disease on Block Island reported in this study and in previous studies [31], Block Island residents were less likely to use protective measures than residents of Connecticut [12], [28], [29]. The most common protective measure was tick checks (53%), followed by wearing protective clothing (44% overall; 46% of seronegative subjects and 28% of seropositive subjects). Only a quarter of the population used any landscape-related control measure, 17% reported using repellents, and 1.5% reported using acaricides. The modest use of protective measures may partially explain the high incidence of Lyme disease on Block Island.

Our study is the first to describe the direct association between landscape structure of individual properties and Lyme disease infection. The landscape metric included in the final model - shrub edge density, was associated with both higher density of nymphs and higher human seropositive rates for Block Island residents. At larger spatial scales, forest fragmentation has been associated with increased tick density and infection prevalence of ticks. Forest fragmentation increases the amount of forest edge and may increase densities of the white-footed mouse (*Peromyscus leucopus*), the most competent host for immature ticks and *B. burgdorferi* [55], [56]. Increased forest edge also has been linked to increased [22] and decreased [21] Lyme disease incidence. Although all shrub-associated landscape metrics were associated with higher density of nymphs, we found that only higher shrub edge density was associated with positive Lyme serology. This metric measures the length of edge *per total property area*, and may result in more frequent residents’ contact with shrub edges – and ticks, compared with properties with more edge but distributed over a larger area. Further research is needed to better understand people’s interaction with their environment to help refine general recommendations [57] that may not be applicable to a specific lifestyle or may be undesirable for environmental or recreational reasons [15].

We used serology as a marker of *B. burgdorferi* exposure because it captures subjects with asymptomatic and symptomatic infection and minimizes inclusion of patients misdiagnosed as having had Lyme disease. While *B. burgdorferi* antibody clears in most people who experience Lyme disease within two years, it may persist for many years in some individuals [59], [60], which motivated our exclusion of subsequent visits after a positive Lyme serology test. Although the use of antibody is an excellent method to assess *B. burgdorferi* exposure status, some cases of Lyme disease may have been missed. The two-tiered assay has low sensitivity (about 50%) in acute sera obtained from patients with early Lyme disease [58]. On the other hand, there is higher sensitivity in convalescent sera of such patients and especially in patients with late Lyme disease infection. We maximized the sensitivity by obtaining sera in mid to late Fall and early Spring, weeks to months following the preceding Lyme disease transmission season.

One of the limitations of our study was our inability to determine the site where exposure occurred. Even though the study was restricted to people who lived on Block Island more than three months during the peak transmission period, island residents might have acquired the infection on the mainland or away from their residence, reducing the expected association between peridomestic risk and infection. Peridomestic exposure has been found to be the main site of acquisition of *B. burgdorferi* infection in the Northeast [24], while recreational exposure has been proposed to mainly account for infection patterns in the Midwest [18]. However, recreational exposure may also occur in the Northeast [61], including Block Island. Further research is needed to quantify the relative roles of these different exposures. An additional limitation was our inability to investigate the variability in the way protective behaviors are used. For example, we did not enquire about the frequency of protective measure use, so we were not able to assess the protective effect that might occur with increasing use.

In conclusion, our results suggest that both environmental and behavioral factors are associated with positive Lyme disease serology. Wearing protective clothing when exposed to tick habitat appears to be the most effective method to reduce exposure to Lyme disease. Employing landscaping strategies which reduce the amount of peridomestic shrub edge density may reduce exposure. Future prospective cohort studies should be conducted to ascertain the interactions between acarological risk, landscape design, and personal protective behaviors in reducing Lyme disease in a community.

## Supporting Information

Figure S1 **Questionnaire for the biannual Block Island serosurveys.**
(DOCX)

Table S1 **Landscape metric description.**
(DOCX)

Table S2 **Land cover classification accuracy assessment.** Producer and user accuracy reflecting errors of omission and commission, respectively, are shown as percent and number of pixels.
(DOCX)
