# Supplementary material for: Integrated Assessment of Behavioral and Environmental Risk Factors for Lyme Disease Infection on Block Island, Rhode Island
Source: PLoS One. 2014 Jan 8;9(1):e84758. doi: 10.1371/journal.pone.0084758 (PMC3885597; doi:10.1371/journal.pone.0084758)
Supplement: Figure S1 — Questionnaire for the biannual Block Island serosurveys. (DOCX) [file pone.0084758.s001.docx]

Figure S1. Questionnaire delivered during the biannual serosurveys

Study #_________

Name _______________________________________Birth date ______________

Permanent address and telephone #_________________________Occupation______________

Vacation street address and telephone # _______________________________________________

______________________________________Fire code # ____________

Have you received the Lyme Vaccine? Yes _______If yes, what year? _______ No_____

Which of these groups’ best describes your ethnic identification? Circle the number of your answer:

1) Asian, 2) Black, not of Hispanic origin, 3) Hispanic, 4) West Indian/Caribbean,

5) White, 6) American Indian, 7) Mixed, 8) Other, 9) Don’t know

**EXPOSURE HISTORY**

**1.** How many years have you spent at your present address (permanent or vacation)? _________

During which months? All ___If not all, check all that apply-

Jan___ Feb___ Mar___ Apr___ May ___ June ___ July ___ Aug ___Sept ___ Oct ___ Nov___ Dec__

**2.**  How many hours a day do you spend out of doors near vegetation?

Less than 1 ___ Several ___ 5 or more ___

**3.** Do you keep a pet? dog___ cat___ horse___ other___

**4.** How frequently do you see deer around your residence?

Daily ___ Weekly ___ Less frequently ___

**5.**  Have you been bitten by a tick this year? Yes ___ No ___

If yes, was it a deer tick ___ wood/dog tick ___ tiny ___ large ___

If yes, was it in your town? ___ Elsewhere? ________________

**6.**  Do your tick bites itch? Yes ___ No ___

**7.** When outdoors, what personal protection measures against ticks do you employ?

None ___ Repellant ___ Long pants/socks___ Avoid brush___Tick check ___

**8.**  Do you try to control ticks around your residence? Yes ___ No ___

Chemical spray ___ Damminix ___ Brush control ___ Other ________

**ILLNESS HISTORY**

**9.**  Have you ever been diagnosed with Lyme disease____ babesiosis ___anaplasmosis____?

If so, by symptoms ___ blood test ___ both ___

When? ________ What was your treatment? _____________

Name of physician _______________________ City ______________

How much did your illness cost you (time lost, physician and treatment costs, etc.)? __

**10.**  Have you had any of the following signs of illness this year?

A) rash B) chills C) fever D)headache E) muscle aches F) fatigue G) night sweats H) joint pains I) swelling J) nasal congestion K) cough L) sore throat

1. How many people do you know who have had Lyme disease? _______
2. Have you ever been diagnosed to have immunodeficiency? _______
3. Have you had problems with recurrent infections in the last 10 years? ______________

**14.** Are you on long-term steroids or other immunosuppressive medication? __________
