# Supplementary material for: Integrated Assessment of Behavioral and Environmental Risk Factors for Lyme Disease Infection on Block Island, Rhode Island
Source: PLoS One. 2014 Jan 8;9(1):e84758. doi: 10.1371/journal.pone.0084758 (PMC3885597; doi:10.1371/journal.pone.0084758)
Supplement: Table S1 — Landscape metric description. (DOCX) [file pone.0084758.s002.docx]

Table S1. Landscape metric description

| **Landscape Metric** | **Description** |
| --- | --- |
|  |  |
| Class Area | Equals the sum of the areas (m^2^) of all patches of the corresponding patch type, divided by 10,000 (to convert to hectares); that is, total class area. |
| Largest Patch Index | Equals the area (m^2^) of the largest patch in the landscape divided by total landscape area (m^2^), multiplied by 100 (to convert to a percentage). In other words, LPI equals the percent of the landscape that the largest patch comprises. |
| Total Edge | TE equals the sum of the lengths (m) of all edge segments involving the corresponding patch type. |
| Edge Density | Equals the sum of the lengths (m) of all edge segments in the landscape, divided by the total landscape area (m^2^), and multiplied by 10,000 (to convert to hectares). |
| Landscape Shape Index | LSI equals .25 (adjustment for raster format) times the sum of the entire landscape boundary and all edge segments (m) within the landscape boundary involving the corresponding patch type, including some or all of those bordering background, divided by the square root of the total landscape area (m). |
