# Supplementary material for: Integrated Assessment of Behavioral and Environmental Risk Factors for Lyme Disease Infection on Block Island, Rhode Island
Source: PLoS One. 2014 Jan 8;9(1):e84758. doi: 10.1371/journal.pone.0084758 (PMC3885597; doi:10.1371/journal.pone.0084758)
Supplement: Table S2 — Land cover classification accuracy assessment. Producer and user accuracy reflecting errors of omission and commission, respectively, are shown as percent and number of pixels. (DOCX) [file pone.0084758.s003.docx]

Table S2. Land cover classification producer and user accuracy (errors of omission and commission, respectively).

| **Class** | **Producer Accuracy**  ***(Percent)*** | **User Accuracy**  ***(Percent)*** | **Producer Accuracy**  ***(Pixels)*** | **User Accuracy**  ***(Pixels)*** |
| --- | --- | --- | --- | --- |
| Lawns | 91.2 | 64.1 | 456/500 | 456/711 |
| Shrubs | 98.2 | 59.2 | 491/500 | 491/829 |
| Paved Roads | 97.8 | 79.5 | 489/500 | 489/615 |
| Coast | 93.0 | 94.1 | 465/500 | 465/494 |
| Ocean | 99.8 | 100 | 499/500 | 499/499 |
| Ponds | 100 | 99.8 | 500/500 | 500/501 |
| Big Lakes | 100 | 100 | 500/500 | 500/500 |
| Shadow | 24.4 | 49.2 | 122/500 | 122/248 |
| Urban | 78.0 | 98.7 | 390/500 | 390/395 |
